# Weight Loss in Animals and Humans Treated with “Weighlevel”, a Combination of Four Medicinal Plants Used in Traditional Arabic and Islamic Medicine

**DOI:** 10.1093/ecam/nen067

**Published:** 2011-06-16

**Authors:** Omar Said, Bashar Saad, Stephen Fulder, Khaled Khalil, Eli Kassis

**Affiliations:** ^1^Antaki Center for Herbal Medicine Ltd, Kfar Kana 16930, Israel; ^2^Research and Development Regional Center, the Galilee Society, and Qasemi Research Center, Al-Qasemi Academic College, P.O. Box 2205, Baga Algharbiya 16930, Israel; ^3^Faculty of Allied Medical Sciences, the Arab American University Jenin, Jenin, Palestine; ^4^Sprunk-Jansen, A/S Strandvejen 100, 2900 Hellerup, Denmark

## Abstract

Weighlevel, a mixture of extract of four plants used in traditional Arabic and Islamic medicine as well as in European herbal medicine, was prepared and assessed for its safety and efficacy in weight loss. Leaves of *Alchemilla vulgaris*, *Olea europaea* and *Mentha longifolia* L., as well as seeds of *Cuminum cyminum*, were used. Cultured human fibroblasts treated with Weighlevel did not exhibit any sign of toxicity as evidenced by lactate dehydrogenase release. These results were confirmed in experimental studies on rats where an LD_50_ of 15.3 g kg^−1^ was observed. Significant antioxidant properties were seen at very low concentrations of Weighlevel (10 *μ*g ml^−1^) as measured by the lipid peroxidation method. Progressive and significant weight loss was observed in chickens given this mixture weekly for 4 weeks compared with controls. Furthermore, a 3-fold increase in the thermogenesis was seen in rat interscapular brown adipose tissue following exposure to different concentrations of Weighlevel extract as determined by measurement of increased oxygen consumption. In addition, a clinical study was carried out among 80 human volunteers with a body mass index (BMI) of 30.67 ± 2.14 kg m^−2^. All 80 subjects were asked to continue their usual diet but to eat only three main meals daily and to take one Weighlevel tablet 30 min before each meal. Fourteen subjects were excluded for not following the protocol, and 66 subjects were all evaluated for efficacy and tolerability of Weighlevel monthly for 3 months. Weighlevel was well tolerated by all subjects, and no side effects were reported. A progressive and significant weight loss was seen in these subjects during the whole study period. Higher levels of weight loss were seen in people with BMI of 25–30 kg m^−2^ (overweight) compared to people with BMI >30 kg m^−2^ (obese). The BMI was reduced after 3 months from 28.5 ± 1.2 and 32.1 ± 1.8 kg m^−2^ to 24.5 ± 1.4 and 27.5 ± 2.2 kg m^−2^ in overweight and obese group, respectively. Results indicate safety, tolerability and efficacy of Weighlevel.

## 1. Introduction

Overweight is a major health challenge in the Western world with serious clinical complications such as type 2 diabetes mellitus, cerebrovascular and ischemic heart diseases. Available pharmacological therapy of obesity is limited to anorexic drugs such as amfepramon and sibutramine and one malabsorptive drug, orlistat. When added to a diet, such therapy is expected to yield weight loss of 0.2–0.4 kg week^−1^, but only for few weeks due to tolerance and side effects [1].

Effective management of overweight seems to be of utmost importance especially if modern science can utilize safe plants derived from traditional medicine to fill the gap and supplement currently used pharmacological products. Mild overweight was generally accepted in ancient Arab societies and considered a reflection of a high socioeconomic level and part of the desired beauty standards in those days. This fact was expressed in the minimal effort to discover anti-obesity remedies compared with other medical fields. Only severe obesity was considered suitable for treatment using specific medicinal plants, body exercises and control of food consumption [2, 3]. Weighlevel is composed of four herbs developed according to the traditional herbal knowledge of the Greek-Arab medical system [4–6]. *Cuminum cyminum L*. (Cumin), *Mentha longifolia L*. (sorting menthe) and *Olea europaea L*. (olive) leaf are all classed as foods or spices throughout Europe and are taken in large amounts, while *Alchemilla vulgaris* L. (lady's mantle) is regarded as safe by the German Commission even at large doses without known adverse effects [7]. Deeply rooted in Arabic medicine, *A. vulgaris* L. has been used for treating obesity, gastrointestinal pain and inflammation [8, 9]. Olive leaves are a typical herbal remedy of the Mediterranean area and reported to possess hypoglycemic, hypotensive-diuretic and antioxidant properties [7, 10]. Extracts from both of these plant leaves have been shown to increase the basal metabolic rate [11, 12]. For centuries, mint as well as cumin has been used to reduce appetite [8] to improve digestion by relieving digestive symptoms such as pain, spasm, gas and dyspepsia and creating a sensation of fullness [13]. The group of carminative seeds such as cumin, caraway, fennel, dill and anise have all been acknowledged to have stomach-calming effects to improve digestion and regulate appetite, especially in children [14].

All four plants are thus used in European herbalism and each with a different indication for use. We hypothesized that these four plants when combined may generate a synergistic effect. The present study aimed to investigate the safety and therapeutic efficacy of a fixed mixture of these four plants. As for safety, we performed *in vitro* and animal studies. As for therapeutic efficacy, we defined it as an incremental and persistent weight loss during the study period of 4 weeks in the controlled animal study and of 3 months in the open human study.

## 2. Materials and Methods

The leaves of lady's mantle, olive and wild mint, as well as the seeds of cumin were collected from the Galilee region, dried under shade and powdered to a fine grade as extracts by Antaki Ltd Laboratories, Kfar Kana, Israel. Weighlevel tablets (310 mg tablet^−1^) were prepared at Karmat Micro Encapsulation laboratories, Kibbutz Ramot Menashe, Israel. Each tablet contained 60 mg *A. vulgaris* L., 50 mg *O. europaea* L., 20 mg *Mentha longiforia* L., 25 mg *C. cyminum* L., 7 mg vitamin C and 148 mg tricalcium phosphate.

### 2.1. Safety and Oxidative Stress Analysis

Rats and rat liver homogenates were used to assess safety and antioxidant effects of Weighlevel.

#### 2.1.1. LD_50_

Thirty-six male Sprague-Dawley rats (average weight: 153 ± 12 g) were divided into four groups. Large single doses of Weighlevel were placed directly into the stomach of each group and observed for 14 days to determine the LD_50_. Animal study approval was given from the Faculty of medical school, Technion, Haifa, in 1999.

#### 2.1.2. Oxidative Stress

Oxidative stress leads to generation of reactive oxygen species (ROS), which play an important pathogenetic role in different disease states. Lipid peroxidation has damaging effects on liver cell membrane. The extent of lipid peroxidation was measured using a technique based on the thiobarbituric acid reactive substance (TBARS) assay that detects malondialdehyde (MDA), an end product of peroxidative decomposition of polyeonic fatty acids in *in vitro* systems [15]. To accurately quantify TBARS in the analytical procedure, the protein was precipitated before the addition of thiobarbituric acid to the reaction, while the antioxidant butylated hydroxytoluene was added before heating of samples. Rat liver homogenates were incubated with 100 *μ*M FeSO_4_ as ROS generating system [16, 17] and with various concentrations of the product.

#### 2.1.3. Lactate Dehydrogenase Assay

Release of the intracellular enzyme lactate dehydrogenase (LDH) is the consequence of necrotic or toxic cell membrane rupture. Integrity of the cell membrane was determined by measuring LDH activity released into the culture medium [18, 19]. LDH activity was monitored following the oxidation of NADH as the decrease in absorbance at 334 nm. The reaction was carried out in a potassium phosphate buffer (40 mM K_2_HPO_4_, 10 mM KH_2_PO_4_, pH 7.5) containing 0.24 mM NADH and 0.62 mM pyruvate. The percentage of LDH released was defined as the ratio of LDH activity in the supernatant to the sum of LDH amount released plus LDH activity measured in the cell lysate. Human fibroblasts were incubated with different amounts of the product extracts, and LDH activity was measured in the medium at 24, 48 and 72 h of incubation.

#### 2.1.4. Thermogenesis

Male Sprague-Dawley rats (*n* = 12) were housed in a light and temperature controlled room (12:12 h light:dark cycle and 23°C) and were given free access to water and a standard laboratory diet for one-week adaptation period, before starting the study. Strips of interscapular brown adipose tissue (IBAT) were rapidly dissected out from the middle part of the fat pad. The tissues were perfused with Krebs-Ringer bicarbonate buffer of the following composition: 116.8 mM NaCl, 25 mM NaHCO_3_, 5.9 mM KCl, 1.2 mM MgSO_4_, 1.2 mM NaH_2_PO_4_, 1.25 mM CaCl_2_ and 5 mM glucose. The Weighlevel extract was added in the perfusion buffer medium, and the resulting suspension was automatically filtered before entering the respiratory chambers. The medium was bubbled continuously with a mixture of 95% O_2_ and 5% CO_2_ [20, 21]. The respiratory rates of IBAT fragments were measured by a method involving repeated O_2_ uptake determinations, as described by Barde et al. [22]. The O_2_ partial pressure (PO_2_) was measured by a Clark O_2_ electrode when extract administration resulted in changes in MO_2_ [23–25].

#### 2.1.5. Efficacy in Chickens

Chickens (average weight around 100 g each) were used in the first phase of our study because they represent a simple and a quick test system. Chicken were cared for in standard conditions and fed for 1 month with normal chick food (*n* = 20), with normal food enriched with 10% *Nigella sativa* (*n* = 20) and with normal food enriched with 3% of the product powder (*n* = 20). Body weight was carefully estimated each week.

#### 2.1.6. Clinical Investigations

Human volunteers were selected on the basis of routine visits to their general physicians in five different clinics in Galilee where they were asked if they were willing to take a herbal therapy for weight loss after a thorough explanation of Weighlevel components. The mean age of 80 subjects recruited into the study was 34.3 ± 9.68 (range 19–53) years. They had an average weight of 90 ± 5 kg and a height of 169 ± 5 cm corresponding to a body mass index (BMI) of 30.67 ± 2.14 kg m^−^^2^. Women comprised 48% of subjects with an age range of 49–67 years. Almost half of all subjects were on some medications mainly for ischemic heart disease, diabetes mellitus and/or hypertension. All medications were kept unchanged during the study period as patients were in a stable clinical condition. Fourteen subjects were excluded as they violated the protocol, eight due to lack of compliance and six due to absence from scheduled visits. Therefore, efficacy and tolerability are given for the remaining 66 subjects. Human volunteers were asked to continue their daily activities and habits, especially their food intake, but to restrict it to three main meals and to remember to take one tablet of Weighlevel 30 min before each meal. Control group was asked to restrict it to three main meals. All usual medications were kept unchanged during the study period of 3 months. An informed consent was obtained from each subject who was given a free-of-charge box containing 90 tablets of Weighlevel. At baseline, the BMI was estimated for every subject. The next visit was scheduled for the coming 4 weeks and they were asked to return the Weighlevel box so that returned tablets could be counted as a measure of patient compliance with the protocol. This was repeated in each of the three consequent visits when body weight was estimated and careful investigations of well-being and of any adverse effect were undertaken.

### 2.2. Statistics

The Wilcoxon signed-rank test was used. Comparisons between groups were performed by the Wilcoxon rank-sum test. A 0.05 level of significance was set. Data obtained were expressed as mean ± SEM.

## 3. Results

### 3.1. Safety Analysis

**Figure 1:**
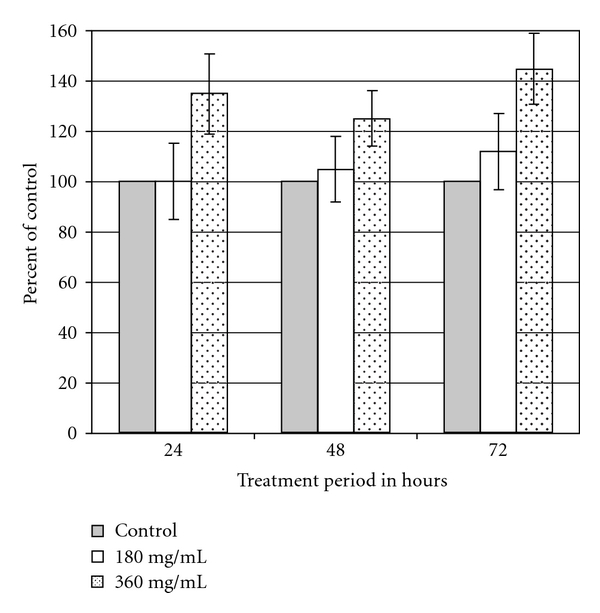
LDH release from cultured human fibroblasts. LDH release was assessed 24, 48, and 72 h in control cells and Weighlevel-treated cells (180 and 360 mg ml^−1^). Values given represent the mean ± SEM of three independent experiments carried out in triplicates.

An extremely high dose of Weighlevel (15.3 g kg^−1^) was necessary to obtain an LD_50_ in rats. On a body weight basis, this would correspond to the human consumption of more than a kilogram. Similar results were seen in *in vitro* studies by measuring the LDH release.  Figure 1 shows the results at 24, 48 and 72 h of incubation. Compared with untreated control cells, no significant change in LDH release was found, whether as a function of increasing product concentration or as a function of increasing incubation period.

### 3.2. Anti-Oxidant

**Figure 2:**
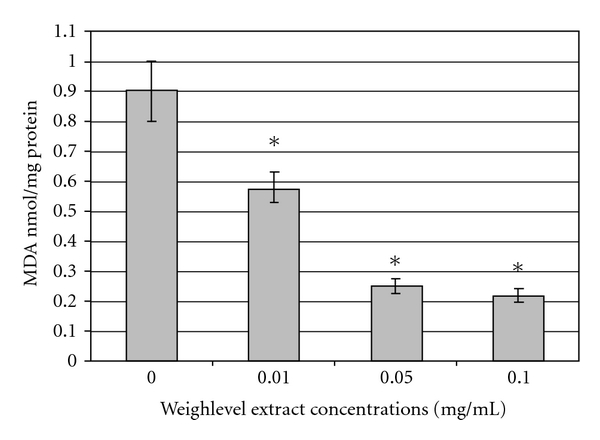
Effects of Weighlevel extract different on MDA release from rat liver homogenates incubated with 100 *μ*M ferrosulfate in the presence and absence of 0.01, 0.05 and 0.1 mg Weighlevel ml^−1^. Values given represent the mean ± SEM of three independent experiments carried out in triplicates.

Lipid peroxidation induced by incubating the rat liver homogenate with ferrosulfate is expressed in Figure 2 as the extent of MDA production. The addition of a very low dose of the product (10 *μ*g ml^−1^) to the medium significantly reduces MDA release from 0.89 ± 0.05 to 0.53 ± 0.03 nM mg^−1^ protein (*P* < .001). Higher concentrations of the product (50 *μ*g ml^−1^) further reduce MDA concentration to 0.28 ± 0.03 nM mg^−1^ protein (*P* < .001). No further anti-oxidative effect of the product is noted by increasing its concentration from 50 to 100 *μ*g ml^−1^ (Figure 2).

### 3.3. Anti-Overweight

**Figure 3:**
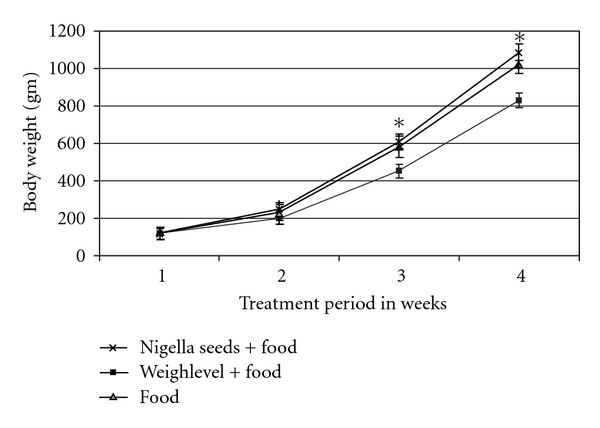
Efficacy of feeding 20 chickens with normal food enriched with 3% of Weighlevel plant extracts (lower diagram) compared with normal food (middle diagram, control group) and normal food enriched with 10% *N. sativa* (upper diagram, positive control group). Body weight values at 1, 2, 3 and 4 weeks are not significantly different in the controls and positive controls, while the studied group had significantly lower values (*P* < .005) at 2, 3 and 4 weeks.

Figure 3 summarizes the controlled efficacy of studies in chickens. Baseline body weight of the 60 chickens was 142 ± 12 g and increased to 1000 ± 15 g at 4 weeks in normal chick feeding. In the positive control group fed with 10% *N. sativa* and normal food, body weight was not substantially higher than that of the control group during the 4 weeks of the study. Significant and incremental reduced weight gain was seen in the study group fed with normal food enriched with 3% of product extract week by week. Body weight in the study group reached 815 ± 10 g at Week 4 (*P* < .005 versus control and versus positive control groups). This reduced weight gain in the study group did not reflect a toxic effect since all three groups were healthy chickens that apparently did not suffer from obesity.

### 3.4. Thermogenesis

**Figure 4:**
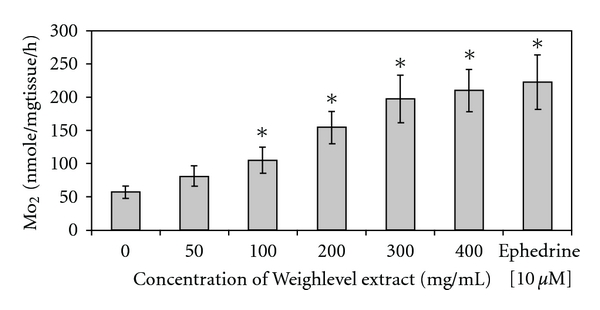
Thermogenesis effects of Weighlevel were determined in strips of rat IBAT. Values given represent the mean ± SEM of three independent experiments carried out in triplicates.

The effects of Weighlevel on thermogeneis are shown in Figure 4. Weighlevel extracts stimulate IBAT respiration rate, in a dose-dependent manner up to >3-fold higher than basal MO_2_ values.

### 3.5. Clinical Investigations

The mean age of 80 subjects recruited into the study was 34.3 ± 9.68 (range 19–53) years. They had an average weight of 90 ± 5 kg and a height of 169 ± 5 cm corresponding to a BMI of 31.3 ± 1.1 kg m^−^^2^. Women comprised 48% of subjects, with an age range of 49–67 years. Almost half of all subjects were on some medications mainly for ischemic heart disease, diabetes mellitus and/or hypertension. All medications were kept unchanged during the study period as patients were in a stable clinical condition. Fourteen subjects were excluded as they violated the protocol, eight due to lack of compliance and six due to absence from scheduled visits. Therefore, efficacy and tolerability are given for the remaining 66 subjects.

**Figure 5:**
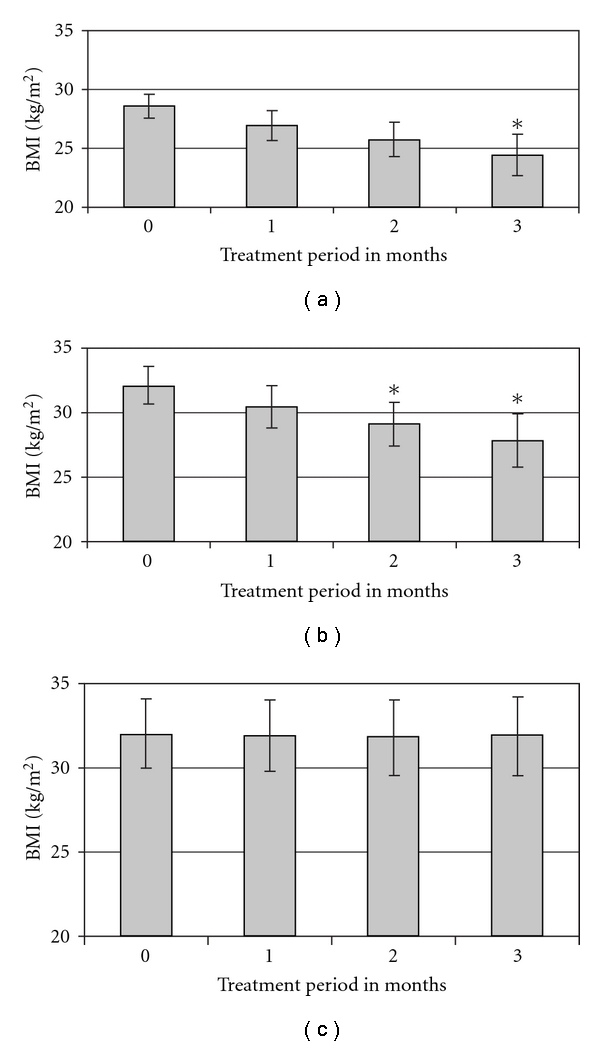
Clinical efficacy studies of Weighlevel in 66 human volunteers who were selected on the basis of routine visits to their general physicians in five different clinics in Galilee where they were asked if they were willing to take an herbal therapy for weight loss. The people were divided into three groups. The first group with BMI of 25–30 kg m^−^^2^ (a), the second group with BMI >30 kg m^−^^2^ (b) and the control group (c). Values given represent the mean ± SEM.

Weighlevel was well tolerated in all 66 subjects and no minor or major adverse effect was noted by any of them. The product was well tolerated with other medications for diabetes mellitus, hypertension, cholesterol and ischemic heart disease.  Figure 5 summarizes the efficacy of Weighlevel in these 66 subjects. Significant and progressive weight reductions were observed each month averaging 1 kg week^−1^ over 3 months. The weight was reduced from baseline of 90.5 ± 1.2 to 78.5 ± 1.4 kg at 3 months (*P* < .0005). A progressive and significant weight loss was seen in these subjects during the whole study period. Higher levels of weight loss were seen in people with BMI of 25–30 kg m^−^^2^ (overweight) (Figure 5(a)) compared with people with BMI >30 kg m^−^^2^ (obese) (Figure 5(b)). The BMI was reduced after 3 months from 28.5 ± 1.2 and 32.1 ± 1.8 kg m^−^^2^ to 24.5 ± 1.4 and 27.5 ± 2.2 kg m^−^^2^ in overweight and obese group, respectively. No significant effects were seen in the control group who was asked to restrict it to three main meals (Figure 5(c)).

## 4. Discussion

The currently practiced traditional Arabic-Islamic herbal medicine is greatly underexploited though it may provide effective new concepts and a rich source of active herbal compounds. According to our knowledge, there are currently no Arab traditional medicine training programs in any Arab country, and Arab medicine has not emerged as a comprehensive health alternative comparable to other non-Western health models. According to recent surveys, more than 2600 plant species are found in the Mediterranean region and *∼*200–250 plants are noted for their uses as medicinal herbs [26–28]. Newly conducted ethnopharmacological survey by our group revealed that more than 450 plant species are still used in our region (Said O., Khalil K. and Saad B., unpublished data). In the present study, we prepared a combination of four herbal remedies that are traditionally known for their weight reduction effects. This combination is called Weighlevel.

The recommended official dose of *A. vulgaris* L., the main medicinal plant in Weighlevel, is 5–10 g day^−1^ of the dried plant [7]. We showed a high level of safety of Weighlevel. Toxicity, as defined by the LD_50_ in rats, was observed only at the high concentrations of *∼*5 g kg^−1^. Concentrations as high as 360 mg ml^−1^ did not show any sign of cellular toxicity in the LDH test. Yet, the antioxidant properties were evidenced at concentrations as low as 0.01 mg ml^−1^ and were more significant at concentrations of 0.05 mg ml^−1^. Higher concentrations did not substantially add to such properties. These observations that show a very high therapeutic index (the difference between effective and toxic doses) may explain the lack of any adverse effect in the studied patients.

The clinical results disclose that Weighlevel is safe and well tolerated by all 66 subjects and is therapeutically efficient as weight loss in each subject was incremental and steady throughout the study period of 3 months.

As experienced in clinical practice, smaller doses of synergistic drugs may yield a better therapeutic efficacy with fewer side effects. Currently, available pharmaceutical drugs when added to a diet are expected to result in weight loss of 0.2–0.4 kg week^−1^ [1]. In the present study, all subjects were asked to continue their usual habits of food intake, but to take only three main meals daily, without snacks in between. We observed weight loss of *∼*1 kg week^−1^ during each of the 12 study weeks, thus reducing the average individual weight by *∼*12 kg during 3 months. The exclusion of 14 subjects was due to the lack of compliance and not due to the lack of efficacy.

As evidenced in the controlled studies in chickens, the reduced weight gain was substantial during 4 weeks in the study group. This could have reflected a toxic effect of Weighlevel in these chickens. All three chicken groups that were fed with the same quality and quantity of normal chick food, were apparently healthy before and during the four study weeks, and each chicken in the three groups normally increased its weight. However, the weight gain in the positive control group was not substantially above that in the control group, whereas it was significantly less in the study group. Therefore, it seems more likely that the observed effects of Weighlevel in these chickens are due to an increased and extended time of satiety and the sensation of fullness along with an augmented basal metabolic rate.

We speculate that the combination of the four plants in Weighlevel acts to increase both satiety and thermogenesis in brown adipocytes by measuring thermogenesis in male Sprague-Dawley rats. This system is generally accepted as a model for fat depletion (fat burning). The amines of *A. vulgaris* L. are mainly the tannins reported to increase the metabolic rate in cold environments [11] and the flavonoids are reported to regulate digestive enzymes and to have cardioprotective effects [29]. Besides metabolic stimulation [12], olive leaf extracts were shown to inhibit intestinal glucose absorption, and a hypoglycemic effect was reported together with hypotensive and hypolipidemic properties [30–32]. Olive leaves are thus known to reduce fat load and circulatory fat levels. Wild mint was reported to relax the stomach and increase gastric emptying and the passage of food through the digestive system [33]. Cumin was also reported to improve glucose utilization, reduce raised blood sugar and promote digestion by stimulating gastrointestinal mucosa and pancreatic digestive enzymes [34, 35].

In addition to the anti-overweight effects of our plant combination, a positive and desired antioxidant activity was observed. This finding is of great importance for people suffering from obesity who usually have high levels of oxidative stress. Based on these findings and taking into consideration the possibility of a tailing off of effectiveness with time, we would encourage further research worldwide, in particular a multi-center open trial in extremely obese subjects. A weight loss of *∼*0–50 kg is to be expected during 1 year of Weighlevel administration three times daily and 30 min before each main meal.
